# Characterization of RanBPM Molecular Determinants that Control Its Subcellular Localization

**DOI:** 10.1371/journal.pone.0117655

**Published:** 2015-02-06

**Authors:** Louisa M. Salemi, Sandra O. Loureiro, Caroline Schild-Poulter

**Affiliations:** Robarts Research Institute and Department of Biochemistry, Schulich School of Medicine & Dentistry, The University of Western Ontario, London, Ontario, Canada; University of Toronto, CANADA

## Abstract

RanBPM/RanBP9 is a ubiquitous, nucleocytoplasmic protein that is part of an evolutionary conserved E3 ubiquitin ligase complex whose function and targets in mammals are still unknown. RanBPM itself has been implicated in various cellular processes that involve both nuclear and cytoplasmic functions. However, to date, little is known about how RanBPM subcellular localization is regulated. We have conducted a systematic analysis of RanBPM regions that control its subcellular localization using RanBPM shRNA cells to examine ectopic RanBPM mutant subcellular localization without interference from the endogenously expressed protein. We show that several domains and motifs regulate RanBPM nuclear and cytoplasmic localization. In particular, RanBPM comprises two motifs that can confer nuclear localization, one proline/glutamine-rich motif in the extreme N-terminus which has a dominant effect on RanBPM localization, and a second motif in the C-terminus which minimally contributes to RanBPM nuclear targeting. We also identified a nuclear export signal (NES) which mutation prevented RanBPM accumulation in the cytoplasm. Likewise, deletion of the central RanBPM conserved domains (SPRY and LisH/CTLH) resulted in the relocalization of RanBPM to the nucleus, suggesting that RanBPM cytoplasmic localization is also conferred by protein-protein interactions that promote its cytoplasmic retention. Indeed we found that in the cytoplasm, RanBPM partially colocalizes with microtubules and associates with α-tubulin. Finally, in the nucleus, a significant fraction of RanBPM is associated with chromatin. Altogether, these analyses reveal that RanBPM subcellular localization results from the combined effects of several elements that either confer direct transport through the nucleocytoplasmic transport machinery or regulate it indirectly, likely through interactions with other proteins and by intramolecular folding.

## Introduction

Transport in and out of the nucleus of proteins above 50KDa is an active process that requires the nucleocytoplasmic transport machinery [1]. Import to the nucleus is mediated by a nuclear localization signal (NLS) that is recognized by an import receptor (importin) which transports its cargo through the nuclear membrane in an energy-dependent process [2]. Conversely, nuclear export is dependent on a nuclear export sequence (NES) that is recognized by exportins, which transport the protein out of the nucleus. Nuclear localization sequences fall into three classes: a short stretch of basic amino acids, a bipartite NLS consisting of two short stretches of basic residues separated by 10–12 amino acids and a combination of charged/polar and non-polar residues flanked by proline and aspartic acid residues [3,4]. The most common characterized NES consists of a non-conserved motif made up of hydrophobic residues and is leucine-rich [2]. Nucleocytoplasmic transport is a tightly monitored process regulated at many different stages [2,3]. One mechanism of regulation includes importin protein expression, as different importins recognize different cargoes. Another mechanism of regulation involves alteration of sequence affinity to karyopherins, for example by phosphorylation of the signal sequence. A third mechanism of regulation involves intermolecular or intramolecular masking of signal sequences. This occurs through protein-protein interactions and conformational changes, respectively, which prevent signal recognition by karyopherins [2,3]. In addition, non-conventional mechanisms exist which do not rely on importins/karyopherins but on interaction with other transporters, or through direct binding to nuclear pore complex components [5].

**Fig 1:**
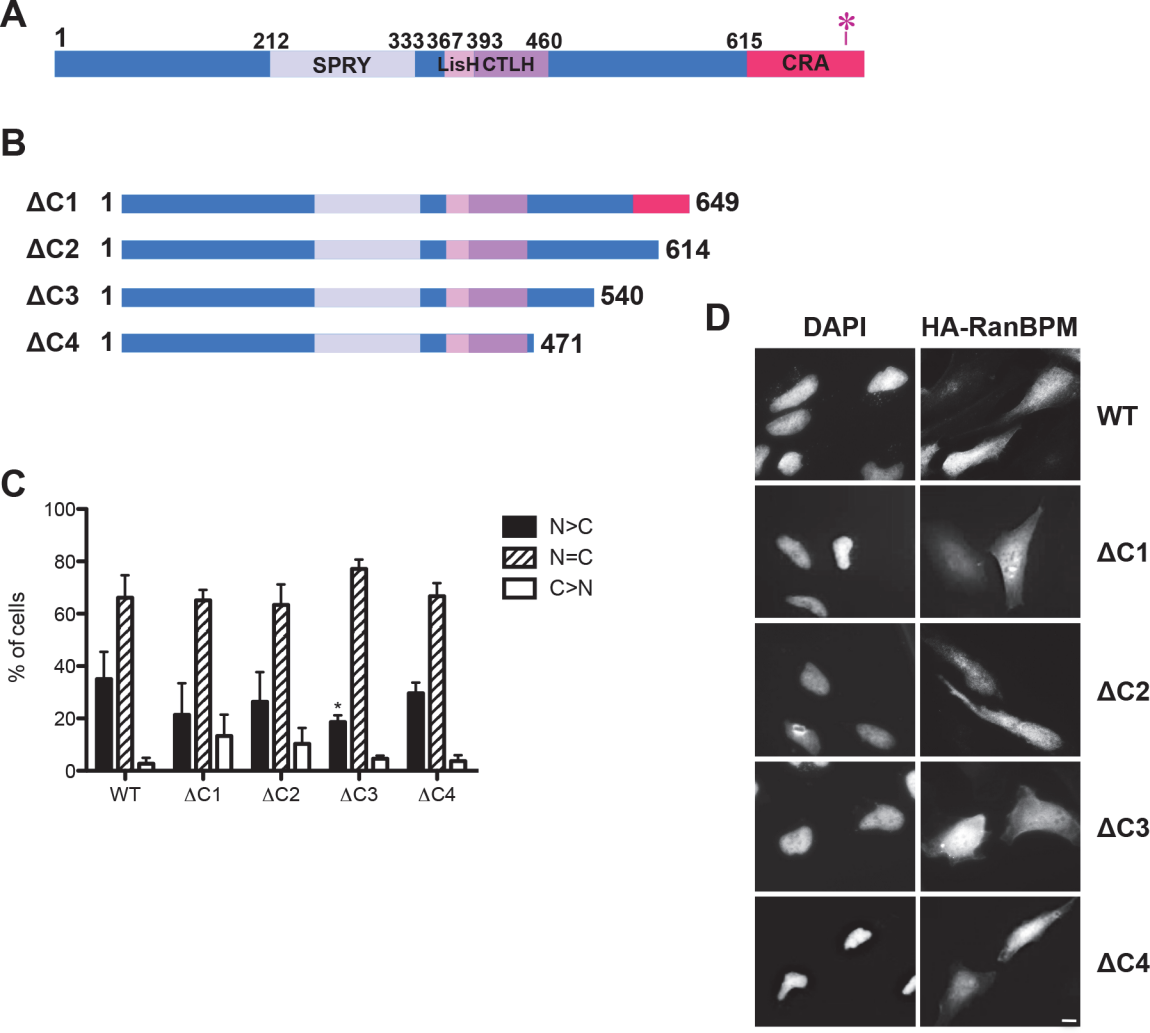
Deletion of RanBPM C-terminus does not alter its subcellular localization. **A)** Schematic diagram of full length wild-type (WT) human RanBPM. The conserved domains are indicated. The red asterisk represents the point mutations conferring siRNA resistance. **B)** Schematic diagram of C-terminal mutant RanBPM constructs. **C)** Analysis of RanBPM deletion mutant subcellular localization. Hela RanBPM shRNA cells fixed 24h after transfection were incubated with an HA antibody and then with an Alexa Fluor 555 secondary antibody. Nuclei were stained with DAPI. Subcellular localization was scored as either, N>C (nuclear greater than cytoplasmic), N = C (nuclear equal to cytoplasmic), or C>N (cytoplasmic greater than nuclear). Data represent averages from three separate experiments, each assessing approximately 100 cells. Error bars represent standard error (SD). Mutant RanBPM constructs versus WT, ***, *P*<0.001; **, *P*<0.01; *, *P*<0.05. **D)** Representative images of transfected mutant RanBPM localization. Scale bar: 10μm.

Ran binding protein M (RanBPM, also referred to as RanBP9), is a ubiquitous, nucleocytoplasmic 90kDa protein whose function is poorly understood. RanBPM contains three conserved domains (Fig. 1A), none of which confers enzymatic activity or is indicative of any specific function, apart from protein interactions. The SPRY (SplA and Ryanodine receptor) domain is a protein interaction domain present in protein families regulating a wide range of functions, including regulation of cytokine signaling, RNA metabolism and protein degradation [6]. The LisH/CTLH (LIS1-homology motif/C-terminal to LisH) domain is found in proteins associated with microtubule dynamics, cell migration and chromosome segregation, and mediates dimerization [7–9]. The CRA (CT11-RanBPM) domain is an α-helical structure of unknown function but is structurally reminiscent of the death domain superfamily [10].

RanBPM has been shown to interact with numerous proteins, implicating it in a variety of cellular processes including cell adhesion, migration, microtubule dynamics, and gene transcription [11–19]. It has been hypothesized that RanBPM functions as a scaffolding protein that may be part of a large complex [20–22]. RanBPM has been identified as a phosphoprotein and its phosphorylation is increased in response to stress stimuli, such as osmotic shock, ultraviolet light (UV), and ionizing radiation (IR) [23,24]. Thus, RanBPM is involved in both nuclear and cytoplasmic processes, but how its subcellular localization is regulated has not been characterized.

RanBPM is well conserved in mammals, in fact the mouse and human proteins are over 90% identical and their differences fall within the N-terminus [20]. The yeast homolog of RanBPM, called Gid1 (glucose-induced degradation-deficient 1) or Vid30 (vacuole import and degradation 30) was found to be part of an E3 ubiquitin ligase complex that functions to ubiquitinate fructose-1,6-bisphosphatase (FBPase), a key enzyme in the gluconeogenesis pathway [25–27]. Recent phylogenetic and sequence analyses revealed that the components of the Gid complex are conserved in eukaryotic genomes, suggesting an ancient and conserved function for this ubiquitin ligase complex in eukaryotes, with RanBPM being one of the most conserved proteins in the complex [28]. In mammalian cells, RanBPM was found in a large cytoplasmic complex together with the mammalian counterparts of all Gid proteins (except Gid4) [22]. This complex was named CTLH complex [22], but is also referred to as the muskelin/RanBPM/CTLH complex (MRCTLH) [28]. The subunits of the complex are present to different extents in both the cytoplasm and the nucleus, yet how their subcellular localization is regulated is still poorly understood [15,21,22,28]. Domain deletion analyses of RanBPM and complex members Twa1, MAEA and RMND5a revealed that several domains in each protein contribute differentially to their localization [28]. Previous investigations showed that the muskelin C-terminal domain is important for both RanBPM interaction and cytoplasmic localization, suggesting that RanBPM regulates the subcellular localization of muskelin [15]. However, how the nucleocytoplasmic localization of RanBPM itself is regulated is still largely unknown.

Here we have carried out a systematic analysis of RanBPM deletion mutants to investigate the determinants of RanBPM subcellular localization. Our results establish that RanBPM subcellular localization is dependent on several domains/motifs, relying on NLS and NES for direct transport by nucleocytoplasmic transport machinery and on protein domains which may function to retain RanBPM to specific subcellular compartments through interaction with other proteins.

## Materials and Methods

### Plasmid Expression Constructs

pCMV-HA-RanBPM shRNA mutant construct (HA-RanBPM si-mt), pCMV-HA-RanBPM-ΔN (ΔN1), pCMV-HA-RanBPM-ΔN2 (ΔN2) and pCMV-HA-RanBPM-ΔC (ΔC1) were previously described [29]. pCMV-HA-RanBPM-Δ212 (Δ212), pCMV-HA-RanBPM-Δ360 (Δ360) and pCMV-HA-RanBPM-ΔC4 (ΔC4) were previously described [30]. pCMV-HA-RanBPM-ΔC2 (ΔC2), pCMV-HA-RanBPM-ΔC3 (ΔC3), pCMV-HA-RanBPM-ΔN3 (ΔN3), pCMV-HA-RanBPM-ΔN4 (ΔN4), pCMV-HA-RanBPM-Δ1-66 (Δ1-66), pCMV-HA-RanBPM-Δ1-25 (Δ1-25) mutant constructs were generated by polymerase chain reaction (PCR) amplification of RanBPM and cloned into digested pCMV-HA-RanBPM si-mt. pCMV-HA-RanBPM-ΔLisH (ΔLisH) and pCMV-HA-RanBPM-ΔCTLH (ΔCTLH) mutant constructs were generated in pCMV-HA-RanBPM si-mt using inverse PCR using tail-to-tail primers on each side of the region to be deleted (367–393 and 393–460, respectively). pCMV-HA-RanBPM-NLS1 (NLS1 Mut), pCMV-HA-RanBPM-NES (NES Mut), and pCMV-HA-RanBPM-NLS2 (NLS2 Mut) point mutations were introduced by site-directed mutagenesis with primers bearing the targeted point mutations. pHM830-WT NLS1/NES, pHM830-MUT NES, pHM830-WT NLS2, pHM830-MUT NLS2, pHM830-1-25, pHM840-WT NLS1/NES, pHM840-MUT NES, pHM840-WT NLS2, pHM840-MUT NLS2, and pHM840-1-25 were produced using annealed oligos that generated overhangs that could be ligated with digested pHM830 and pHM840 ([31], obtained from Addgene). pHM830-LisH/CTLH and pHM840-LisH/CTLH was generated by PCR amplification of RanBPM (aa 360–460) digested and ligated with digested pHM830 and pHM840. All PCR reactions were done using PfuTurbo from Agilent Technologies (Mississauga, ON, Canada) or KOD polymerase (Novagen, Germany) and primers from Sigma-Aldrich (Oakville, ON, Canada), BioCorp UWO Oligo Factory (London, ON, Canada) and Integrated DNA Technologies (Coralville, Iowa, USA).

### Cell Culture, Transfections and Treatments

Hela control shRNA and RanBPM shRNA stable cell lines (2–7 and 2–6) were described previously [29,32] and were cultured in high glucose Dulbecco’s modified Eagle’s medium (DMEM) supplemented with 10% fetal bovine serum (FBS) and 0.35 mg/ml G418 (Geneticin, Bioshop Canada, Burlington, ON, Canada) at 37°C in 5% CO_2_. 3T3 mouse embryonic fibroblasts (MEFs) were cultured in high-glucose DMEM supplemented with 10% FBS. Plasmid transfections were carried out with ExGen500 (Fermentas, Burlington, ON Canada), TurboFect Transfection Reagent (Thermo Fisher Scientific, Burlington, ON, Canada) or jetPRIME (Polypus Transfection) according to the manufacturer’s protocol. Leptomycin B (LMB, Bioshop Canada, Burlington ON, Canada) was added to the cells’ media at 20nM concentration for the times indicated in the figure legends. Nocodazole (Abcam) was added to the cell’s media at 10μM for 4 hours as previously described [33,34].

### Extract preparation, subcellular fractionation, western blot and immunoprecipitations

Whole cell extracts were prepared as described [29] and resolved by SDS-PAGE (between 8% and 12%). Subcellular fractionation and chromatin extractions were adapted from [35]. Briefly, cells were washed twice in PBS, and lysed in buffer A (10 mM HEPES buffer (pH 7.9), 5 mM MgCl, 1 mM DTT, 1 mM phenylmethylsulfonyl fluoride (PMSF), 1 μg/ml of aprotinin, 10 μg/ml of peptatin, 1 μg/ ml of leupeptin and 10 mM KCl), for 25 min and centrifuged at 1000 rpm for 5 min at 4°C and the supernatant (cytosolic fraction) was collected. The pellet was washed in PBS, lysed in buffer B (20 mM HEPES buffer (pH 7.9), 25% glycerol, 5 mM MgCl, 0.2% NP-40, 1 mM DTT, 1 mM PMSF, 1 μg/ml of aprotinin, 10 μg/ml of pepstatin, 1 μg/ ml of leupeptin and 150 mM KCl) for 15 min and centrifuged at 3000 rpm for 5 min at 4°C to collect the supernatant (soluble nuclear fraction). The resulting pellet was washed in PBS and lysed in buffer C (20 mM HEPES buffer (pH 7.9), 25% glycerol, 5 mM MgCl, 0.2% NP-40, 1 mM DTT, 1 mM PMSF, 1 μg/ml of aprotinin, 10 μg/ml of peptatin, 1 μg/ ml of leupeptin and 420 mM KCl) for 15 min and centrifuged at 14000 rpm for 10 min at 4°C, to yield the supernatant which is the chromatin-associated fraction. Samples were resolved on 10% SDS-PAGE and transferred on polyvinylidene difluoride (PVDF) membranes. Samples were analyzed with the following antibodies: HA (HA-7, Sigma-Aldrich), β-actin (I-19, Santa Cruz, Santa Cruz, CA, USA), RanBPM (5M, Bioacademia, Japan), α-tubulin (Sigma-Aldrich), α-tubulin (ab15246, Abcam) and Ku70 (N3H10, Santa Cruz, CA USA). Quantifications were done using ImageJ software. Co-immunoprecipitation experiments were performed in 0.5% NP-40 and 100mM KCl lysis buffer and were carried out overnight at 4°C with α-tubulin (Sigma-Aldrich). Immunoprecipitates were isolated with Dynabeads protein G (Invitrogen, Life Technologies, Burlington, ON, Canada).

### Immunofluorescence

Cells were plated on coverslips and transfected following overnight incubation. Cells transfected with pHM830 and pHM840 vectors were fixed with 3% paraformaldehyde and mounted with DAPI (Invitrogen). Cells transfected with pCMV-HA-RanBPM constructs were fixed with 3% paraformaldehyde, permeabilized in 0.5% Triton-X100 for 10 min and pre-blocked in 5% FBS diluted in PBS. Coverslips were incubated overnight with primary antibodies (see below), washed in PBS and incubated with secondary antibodies: anti-goat Alexa Fluor 488, anti-mouse Alexa Fluor 488, anti-mouse Alexa Fluor 555, anti-rabbit Alexa Fluor 647 or anti-mouse Alexa Fluor 647 (Invitrogen). Cells were mounted with ProLong Gold antifade with DAPI (Invitrogen). Visualization was done using an Olympus BX51 microscope with a 40x objective and images were captured with the Image-Pro Plus software (Media Cybernetics Inc., Bethesda, MD, USA). Primary antibodies used in immunofluorescence: RanBPM (Ab5295, Abcam and K-12, Santa Cruz), HA (HA-7, Sigma-Aldrich), Cyclin B1 (Cell Signaling) and α-tubulin (Sigma-Aldrich). For quantification analysis, images were blinded by a third party and coded images were scored independently by two individuals. For each treatment, at least 100 cells (for HA-RanBPM mutant analyses) or 50 cells (for pHM830/pHM840 analyses) per sample were scored by each individual and results were averaged from at least three separate experiments. Quantitative subcellular localization was performed using ImageJ. Whole cell and nuclear fluorescence signal intensity was measured and subtracted to calculate cytoplasmic fluorescence signal intensity. Nuclear and cytoplasmic intensity was calculated as a percent of whole cell intensity. Confocal images were acquired using an inverted IX51 Olympus microscope equipped with a Perkin Elmer Spinning Disk Confocal attachment with a 60x objective using Velocity software and image analyses were done using Imaris software (Bitplane, Zurich, Switzerland).

### Statistical analyses

Differences between multiple groups were compared using analysis of variance (ANOVA) and differences between two groups were compared using unpaired two-tailed t test. Results were considered significant when *P* <0.05.

## Results

### Analysis of RanBPM deletion mutants

**Fig 2:**
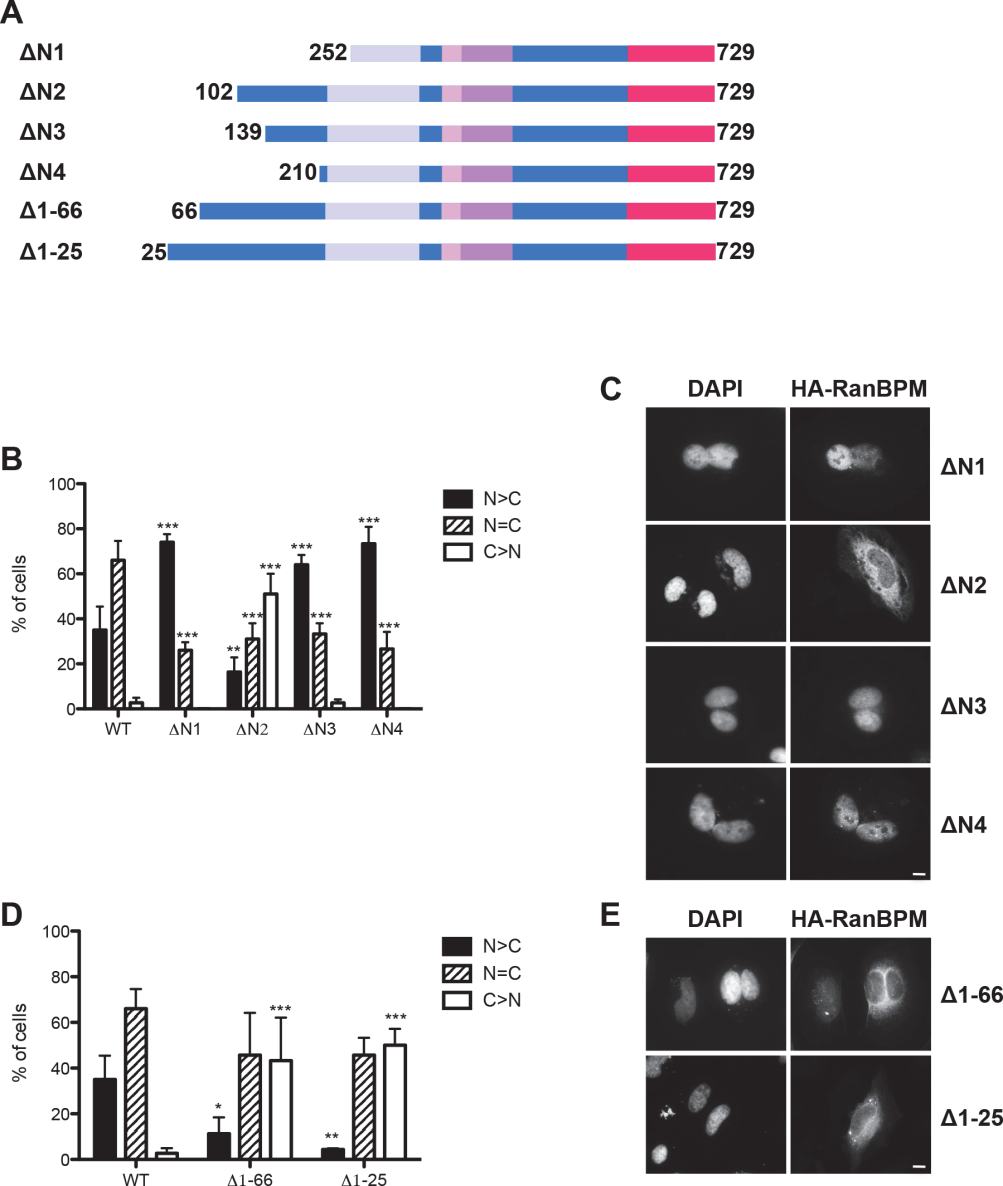
RanBPM N-terminal region contains several determinants that regulate its subcellular localization. **A)** Schematic diagram of N-terminal mutant RanBPM constructs. **B)** Mutant RanBPM (ΔN1-4) were transfected and scored as described in Fig. 1. Data represent averages from three separate experiments, each assessing a minimum of 100 cells. Error bars represent SD. Mutant RanBPM constructs versus WT, ***, *P*<0.001; **, *P*<0.01; *, *P*<0.05. **C)** Representative images of transfected mutant RanBPM localization quantified in B. **D)** Identification of a nuclear targeting sequence in the extreme N-terminal region. Mutants with various deletions of N-terminal sequences (as indicated) were transfected and processed as above. **E)** Representative images of the localization of the transfected mutant RanBPM quantified in D. Scale bar: 10μm.

**Fig 3:**
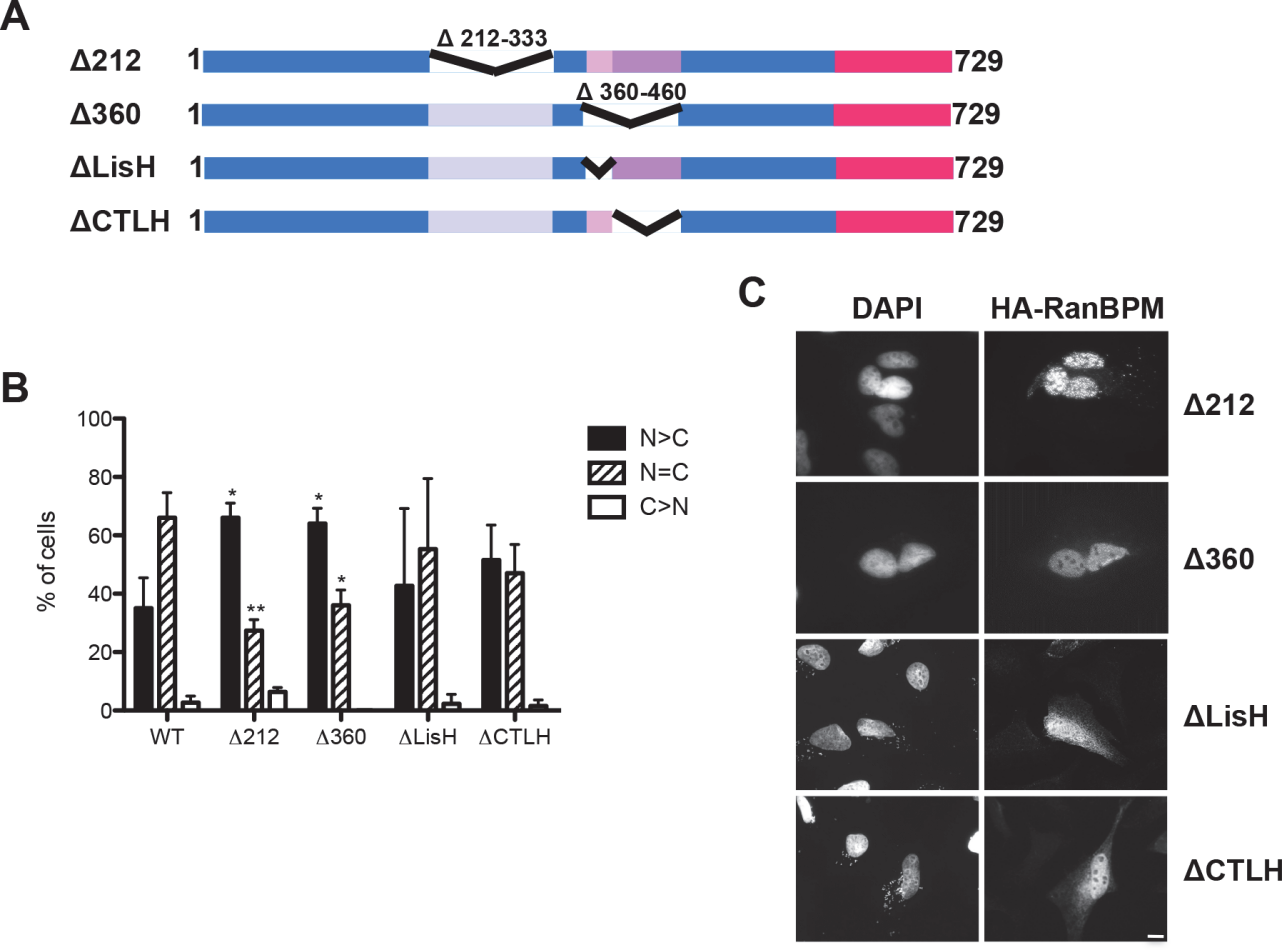
Deletion of RanBPM SPRY and LisH/CTLH domains promotes RanBPM nuclear localization. **A)** Schematic diagram of internal deletion mutant RanBPM constructs. **B)** Cells were fixed 24h after transfection of the RanBPM mutants indicated and incubated with an HA antibody and then with an Alexa Fluor 555 secondary antibody. Nuclei were stained with DAPI. Subcellular localization was scored as either, N>C (nuclear greater than cytoplasmic), N = C (nuclear equal to cytoplasmic), or C>N (cytoplasmic greater than nuclear). Data represent averages from three separate experiments, each assessing approximately 100 cells. Error bars represent SD. Mutant RanBPM constructs versus WT, ***, *P*<0.001; **, *P*<0.01; *, *P*<0.05. **C)** Representative images of transfected mutant RanBPM localization. Scale bar: 10μm.

To start evaluating the regions of RanBPM that regulate its subcellular localization, we engineered a series of deletion mutants lacking N-terminal, C-terminal and internal domain regions (Figs. 1–3). All constructs contained an N-terminal HA tag to assess expression and subcellular localization by indirect immunofluorescence. Mutants were transiently transfected in Hela cells stably expressing a RanBPM shRNA (clone 2–7), in which we have previously shown that RanBPM expression is effectively downregulated to near undetectable levels [29,32,36]. The design of this strategy was prompted by the fact that previous studies have documented that the LisH domain can mediate protein dimer and tetramer formation [7–9]. Indeed a recent report suggested that RanBPM is able to form homo-dimeric or -multimeric complexes [37]. We reasoned that if this were to occur, the RanBPM mutants that retain the LisH domain would not show a substantial change in subcellular localization upon transfection in normal Hela cells as they would be localized based on their interaction with endogenous RanBPM. Thus, expressing the RanBPM mutants in cells lacking endogenous RanBPM would circumvent this possible limitation and also minimize potential artefacts arising from overexpression of the RanBPM protein. To prevent degradation of the transfected constructs by the RanBPM siRNA (which targets a specific sequence located in the extreme C-terminal region, Fig. 1A), all mutants containing the C-terminal region comprised a point mutation in the sequence targeted by the siRNA, as previously described [29].

**Fig 4:**
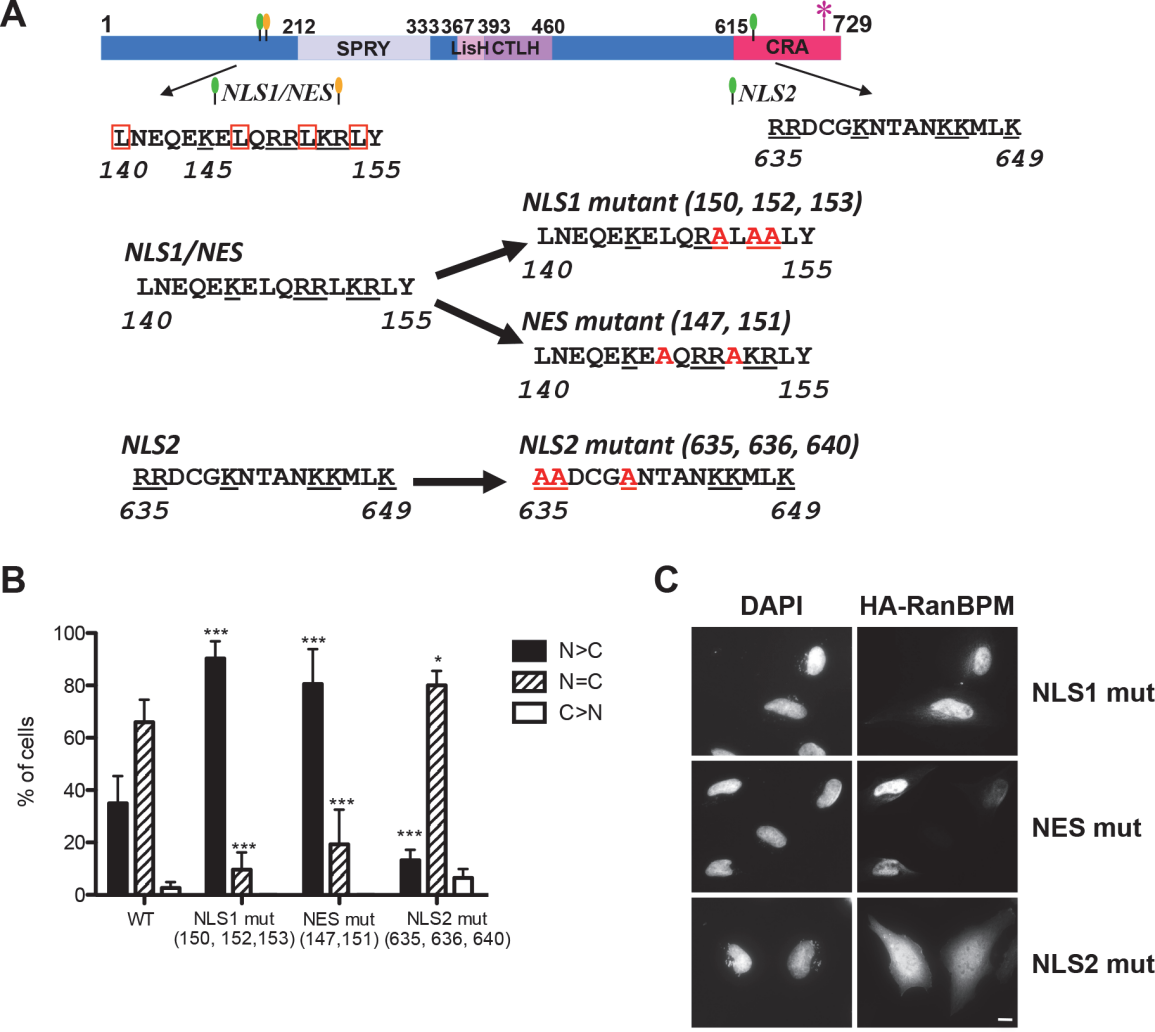
Identification of RanBPM NLS and NES. **A)** Schematic diagram indicating the position of RanBPM putative NLS and NES which are represented by green and yellow flags, respectively. The amino acid sequence of these elements is indicated below, with the WT sequences on the left, and the mutated sequences on the right. Conserved basic residues are underlined, the leucines present in the putative NES are boxed and the alanine point mutations are marked in red. The asterisk represents the point mutations conferring siRNA resistance. **B)** Cells were transfected with the RanBPM mutants indicated and immunofluorescence and scoring was performed as described in Fig. 1. Data represent averages from three separate experiments, each assessing approximately 100 cells. Error bars represent SD. Mutant RanBPM constructs versus WT, ***, *P*<0.001; **, *P*<0.01; *, *P*<0.05. **C)** Representative images of transfected mutant RanBPM localization. Scale bar: 10μm.

To quantify RanBPM localization through indirect immunofluorescence, we employed a localization scoring protocol that we have described previously [29]. Using this approach, RanBPM full-length (wild-type, WT) expressed in RanBPM shRNA cells was determined to present nuclear and cytoplasmic distribution similar to what has been reported for endogenous RanBPM (Fig. 1) [29]. To verify the accuracy of our scoring evaluation, we repeated our measurements of nucleocytoplasmic distribution by quantifying the signal intensity of the whole cell and the nuclear compartment and calculating the resulting cytoplasmic intensity (S1A Fig.). This yielded a similar distribution for WT RanBPM and two RanBPM mutants (described later in Figs. 2 and 4).

Progressive deletion of the C-terminal region (ΔC1-C4) did not alter this nuclear and cytoplasmic distribution, suggesting that the C-terminal region does not primarily contribute to RanBPM subcellular localization (Fig. 1).

N-terminal deletions revealed to have a much greater impact on regulating RanBPM localization. First, a large (251 aa) N-terminal deletion (ΔN1) resulted in a near complete relocalization of RanBPM to the nucleus (Fig. 2B,C). Smaller deletions resulting in truncation of the first 138 and 209 aa (ΔN3 and ΔN4) had a similar effect. However, deletion of the first 101 aa (ΔN2) had a completely reverse effect, resulting in near complete cytoplasmic localization (Fig. 2B,C). This suggested that while the first 101 aa contain sequences required for RanBPM nuclear localization, elements in RanBPM sequences C-terminal to aa 251 can also promote nuclear localization. Interestingly, the region included between aa 102 and aa 138 appeared to contain elements that retain or promote RanBPM localization to the cytoplasm, since its deletion elicited RanBPM relocalization to the nucleus.

To further investigate which region between aa 1 and 102 is responsible for nuclear localization, smaller deletions within this region were generated (Fig. 2). Deletion of the first 66 amino acids (Δ1-66) resulted in an increased cytoplasmic localization similar to that of RanBPM ΔN2, suggesting that nuclear localization determinants were located in the very N-terminal region of the protein (Fig. 2D, E). A smaller deletion of the first 25 amino acids (Δ1-25) still resulted in decreased nuclear and increased cytoplasmic localization, suggesting that the first 25 amino acids contain determinants that direct or retain RanBPM in the nucleus.

We next examined the potential contribution of the SPRY and LisH/CTLH domains to RanBPM subcellular localization. Internal deletion of the SPRY domain (Δ212) resulted in increased nuclear localization compared to WT suggesting that this region plays a role in cytoplasmic targeting of the protein (Fig. 3). The SPRY domain is known to mediate protein-protein interactions [6], suggesting that interaction of RanBPM through this domain with yet unidentified partner(s) may be responsible for cytoplasmic retention. In addition, and in contrast to full length RanBPM and all other mutants examined so far which showed diffuse staining in both the nucleus and cytoplasm, most cells expressing RanBPM Δ212 displayed a speckled nuclear staining, with small aggregates present throughout the nucleus. These aggregates could be the result of misfolding, indicating that this domain may also be needed for correct folding of the protein.

Deletion of the LisH/CTLH domains (Δ360) also resulted in predominant nuclear redistribution of RanBPM, suggesting that this region promotes cytoplasmic localization. Individual deletions of either the LisH or the CTLH domain did not significantly alter RanBPM nucleocytoplasmic distribution (Fig. 3B,C). Altogether, it appears that disruption of both the LisH and CTLH domains perturbs RanBPM subcellular localization by promoting its recruitment to the nucleus.

To confirm that the localization of the RanBPM mutants was not influenced by cell-specific properties of the clonal cell line expressing RanBPM shRNA, the subcellular localization of RanBPM WT, ΔN2 and Δ360 was assayed in another RanBPM shRNA cell line (RanBPM shRNA 2–6). The results revealed no differences in the subcellular localization of these mutants between the two clonal cell lines (S1B Fig.).

### Identification of RanBPM NLS and NES motifs

RanBPM deletion mutant analyses indicated that several regions of RanBPM are involved in regulating its subcellular localization. The RanBPM C-terminal deletions did not affect RanBPM nucleocytoplasmic distribution, which initially suggested that this region did not contain elements contributing to the localization of the protein. However, intriguingly, various deletions of the N-terminal region (ΔN1, ΔN3, ΔN4) caused the relocalization of RanBPM to the nuclear compartment (Fig. 2). This suggested that the C-terminal region contains elements directing RanBPM in the nucleus, even though these may be subordinate to primary nuclear localization signals present in the N-terminus. A search for clusters of basic residues (which are typical of NLS) revealed two potential NLS for RanBPM, in the N-terminus (NLS1, 140–155) and in the C-terminus (NLS2, 635–649) (Fig. 4A). Interestingly, the N-terminal potential NLS1 also features characteristics of a leucine-rich NES, suggesting that this element could be conferring both import and export properties, which has been previously reported for similar sequences [38,39].

**Fig 5:**
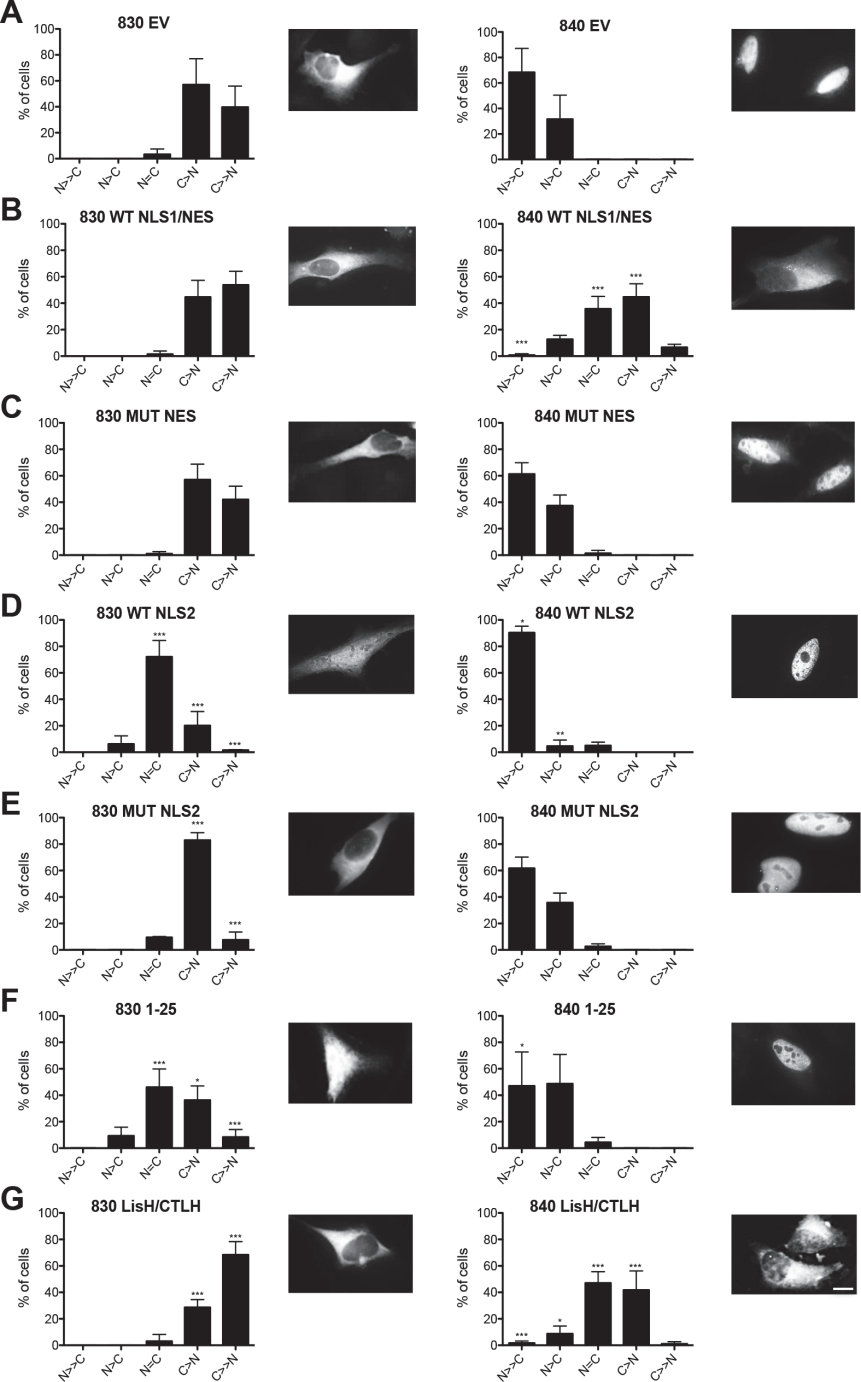
Characterization of RanBPM motifs that confer nuclear or cytoplasmic localization of GFP-β-gal. **A-G)** RanBPM shRNA Hela cells were transfected with either pHM830 (830) or pHM840 (840) empty vectors (EV) or vectors containing various motifs or domains of RanBPM fused to GFP-β-gal. The identity of the RanBPM motif/domain fused to GFP-β-gal is indicated above each panel. *Left*, pHM830 fusion constructs, *right*, pHM840 fusion constructs. Cells were fixed 24 hours after transfection and nuclei stained with DAPI. GFP-β-gal subcellular localization was scored as either N>>C (completely nuclear), N>C (nuclear greater than cytoplasmic, N = C (nuclear equal to cytoplasmic), C>N (cytoplasmic greater than nuclear, or C>>N (completely cytoplasmic). Data represent averages from three separate experiments, each assessing a minimum of 50 cells. Error bars represent SD. RanBPM motifs versus EV, ***, *P*<0.001; **, *P*<0.01; *, *P*<0.05. Inset, representative images of transfected pHM830 or 840 fusion constructs alone (EV) or subcloned with RanBPM motifs. Scale bar: 10μm.

Point mutations of residues assumed to confer either nuclear (K/R) or cytoplasmic (L) localization in the putative NLS1/NES (NLS1 mut and NES mut) in the N-terminal region of the protein resulted in both cases in near complete relocalization of RanBPM to the nucleus, suggesting that this element functions as a NES (Fig. 4B,C). Introduction of point mutations in the putative C-terminal NLS (NLS2 mut) resulted in a slight, albeit significant decrease in nuclear localization (Fig. 4B,C). However this mutant still displayed predominant nucleocytoplasmic localization, suggesting that this element only partly contributes to RanBPM nuclear targeting. To further characterize these motifs, we investigated whether they were able to confer specific localization to a Green Fluorescent Protein-β-galactosidase (GFP-β-gal) fusion protein. For these experiments, we subcloned RanBPM motifs in the pHM830 and pHM840 vectors which encode a GFP-β-gal fusion protein. When expressed from pHM830 (830 EV), GFP-β-gal is cytoplasmic as its size precludes passive diffusion to the nucleus, but addition of a Simian virus 40 (SV40) NLS sequence in the pHM840 (840 EV) vector promotes its localization to the nucleus [31] (Fig. 5A). Fusion of the RanBPM NLS1/NES sequence in pHM830 (830 WT NLS1/NES) did not affect GFP-β-gal cytoplasmic localization (Fig. 5B). However, NLS1/NES was able to promote cytoplasmic export of the nuclear GFP-β-gal expressed from pHM840 (840 WT NLS1/NES), and this was prevented by point mutations in this element (MUT NES, Fig. 5C), confirming that this element functions as a NES. Interestingly, the NLS2 sequence in the C-terminal region of RanBPM was able to promote nuclear localization of cytoplasmic GFP-β-gal (Fig. 5D), and this was prevented by point mutations of three basic residues (MUT NLS2, Fig. 5E). This suggested that this element can function as a NLS, even though its deletion or mutation in the context of the RanBPM protein only mildly affects RanBPM recruitment to the nucleus.

Consistent with the results of the RanBPM deletion mutant experiments, RanBPM 1–25 was able to direct GFP-β-gal to the nucleus, thus suggesting the presence of a nuclear targeting element in this region (Fig. 5F). Altogether, these analyses suggest that RanBPM possesses two elements capable of conferring nuclear localization. However, only the 1–25 NLS appears to efficiently function to direct RanBPM in the nucleus. While NLS2 has the properties of a NLS, it appears to be marginally functional in the context of the full-length RanBPM protein.

Finally, the RanBPM LisH/CTLH domain was able to prevent nuclear localization of the nuclear GFP-β-gal expressed from pHM840 (840 LisH/CTLH), suggesting that the LisH/CTLH domain promotes localization to the cytoplasm, possibly by conferring cytoplasmic retention (Fig. 5G).

**Fig 6:**
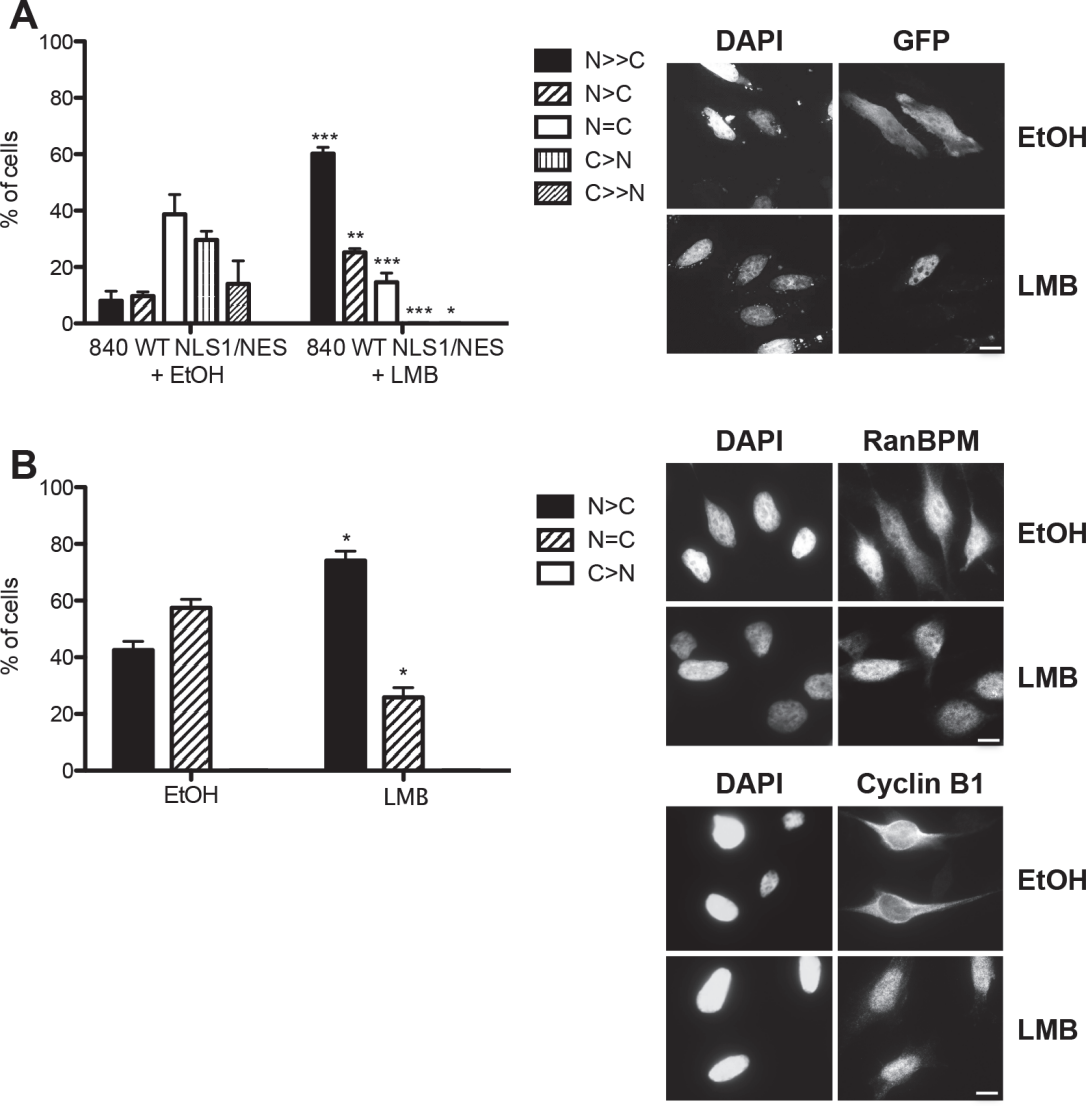
Effect of Leptomycin B (LMB) treatment on RanBPM nuclear export. **A)** RanBPM shRNA Hela cells transfected with 840 WT NLS1/NES were treated with ethanol (EtOH) or 20nM LMB, fixed following 3h of treatment and stained with DAPI. Subcellular localization was scored as either N>>C (completely nuclear), N>C (nuclear greater than cytoplasmic, N = C (nuclear equal to cytoplasmic), C>N (cytoplasmic greater than nuclear, or C>>N (completely cytoplasmic). Data represent averages from three separate experiments, each assessing approximately 50 cells. Error bars represent SD. ***, *P*<0.001; **, *P*<0.01; *, *P*<0.05. **B)** Hela cells were treated with EtOH or 20nM LMB and incubated for 16h. Cells were fixed and processed for immunostaining with antibodies to RanBPM and cyclin B1 and nuclei stained with DAPI. At least 100 cells were scored as N>C (nuclear greater than cytoplasmic), N = C (nuclear equal to cytoplasmic), or C>N (cytoplasmic greater than nuclear). Data represent averages from three separate experiments, each assessing approximately 100 cells. Error bars represent SD. Scale bar: 10μm.

These results suggested a very complex regulation of RanBPM cytoplasmic localization since deletions of the SPRY and the LisH/CTLH domains as well as mutation of the NES sequence all resulted individually to a relocalization of RanBPM to the nucleus. To determine whether RanBPM cytoplasmic localization was subject to a CRM1-export dependent regulation, we treated cells with leptomycin B (LMB) to inhibit CRM1-dependent nuclear export. Surprisingly, standard LMB treatment (20nM, 3h) had no effect on RanBPM localization, while it significantly affected cyclin B1, which undergoes LMB-sensitive nuclear export [40] and accumulated in the nucleus under the same conditions (S2 Fig.). However, treatment of 840 WT NLS1/NES with LMB prevented cytoplasmic accumulation of GFP-β-gal and resulted in its nuclear localization, which suggested that the RanBPM NES activity in this context is sensitive to CRM1 inhibition (Fig. 6A). One possibility to explain these seemingly contradictory results is that only a small fraction of RanBPM is shuttling and thus longer treatment would be needed to reach a detectable accumulation in the nucleus. Thus, we tested the effect of prolonged LMB treatment on endogenous RanBPM localization and found that 16h incubation with LMB induced a significant nuclear accumulation of RanBPM (74.1%) when compared to vehicle treatment (42.6%) (Fig. 6B). These results suggest that RanBPM undergoes CRM1-dependent nuclear export but that the activity of the NES is limited in the context of the RanBPM protein possibly due to the fact that a large proportion of the RanBPM cellular pool is not actively shuttling. Altogether, it appears that RanBPM cytoplasmic localization is dependent on the integrated action of several domains and sequences and that the function of these regulatory regions may be modulated by specific RanBPM protein folding which remains uncharacterized.

### Effect of RanBPM deletions on protein expression

**Fig 7:**
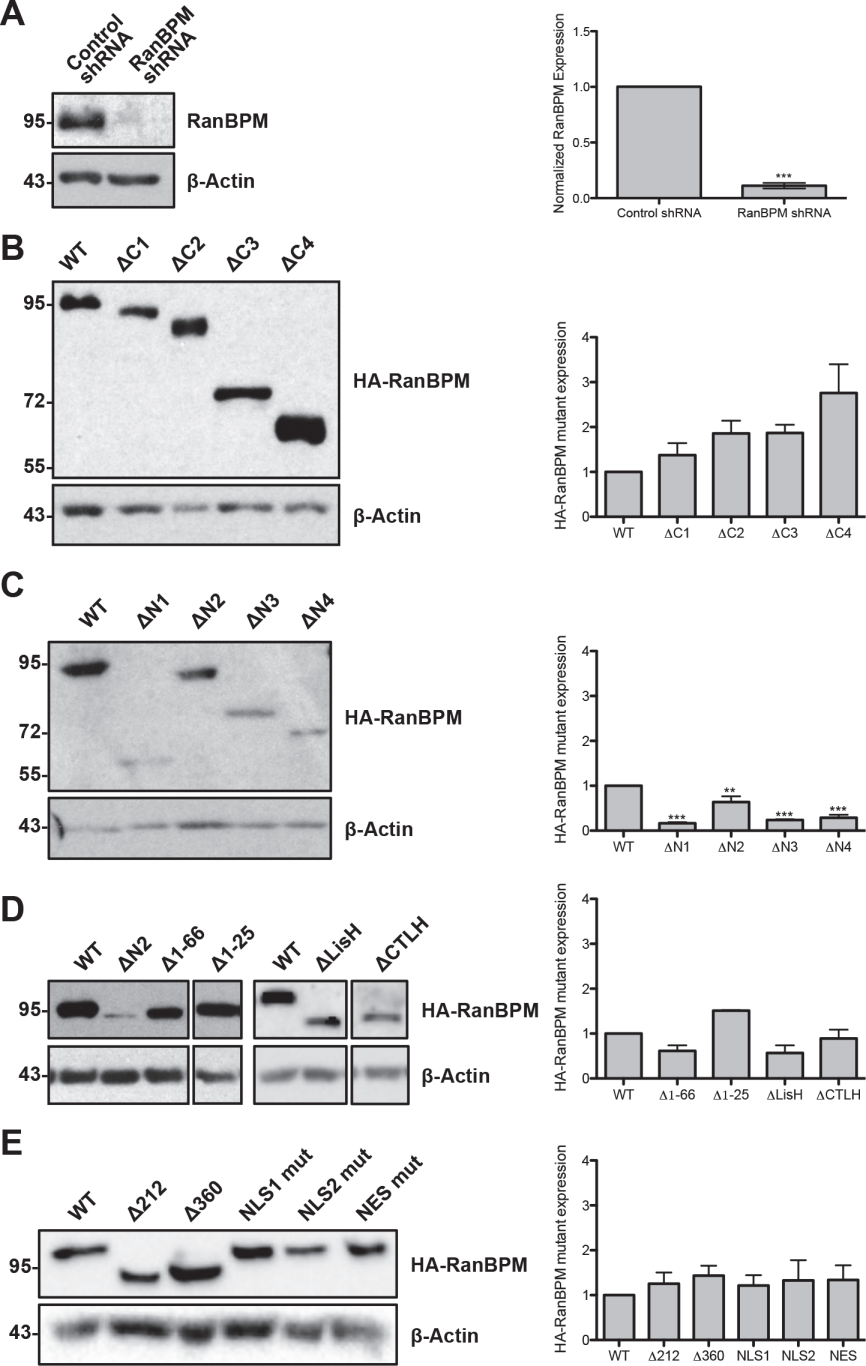
Representative Western blot analysis of endogenous RanBPM in control and RanBPM shRNA cell lines and RanBPM mutant protein expression. **A)** *Left*, Whole cell extracts prepared from control and RanBPM shRNA cells were analyzed by western blot with a RanBPM antibody. *Right*, quantification of endogenous RanBPM normalized to β-actin loading control. Data represent averages from three experiments. Error bars represent standard error. **B)** Expression of wild-type (WT) and ΔC deletion constructs. *Left*, whole cell extracts were prepared from RanBPM shRNA Hela cells transfected with pCMV-HA-RanBPM WT and mutant constructs 24h after transfection. An HA antibody was used to detect HA-RanBPM and a β-actin antibody was used as a loading control. *Right*, quantification of pCMV-HA-RanBPM constructs normalized to β-actin loading control. Data represent averages from three experiments. Error bars represent standard error. Mutant RanBPM constructs versus WT, ***, *P*<0.001; **, *P*<0.01; *, *P*<0.05. **C)** Expression of RanBPM ΔN deletion constructs. Analysis and quantifications are as described above. **D)** Expression of RanBPM Δ1-66, Δ1-25, ΔLisH and ΔCTLH. Analysis and quantifications as described in B. **E)** Expression of Δ212, Δ360 and point mutation constructs. Analysis and quantifications as described in B.

To assess the effect of RanBPM deletions on protein expression, we analyzed the levels of expression of all mutants by western blot. As previously reported, endogenous RanBPM expression is severely reduced in RanBPM shRNA cells (Fig. 7A) [29,32,36]. RanBPM mutants bearing either C-terminal deletions or internal deletions of the SPRY and LisH/CTLH domains were found expressed at levels similar to WT (Fig. 7B,D,E). However, N-terminal mutants showed significantly reduced expression levels, particularly the ΔN1, ΔN3 and ΔN4 deletion mutants, while ΔN2 expression was somewhat decreased (Fig. 7C). It should be noted that, as reported previously for ΔN1 and ΔN2 [29], the expression levels of the ΔN1-4 mutants in individual cells was not noticeably different than WT RanBPM or any other mutant and this is reflected by the fact that exposure times for image capture was similar for all mutants. However, we consistently obtained a lower number of cells transfected with the ΔN1-4 mutants compared to the other mutants, which explains the lower level of expression of these mutants observed by western blot analysis, although the reason for this phenomenon remains unclear. As all mutants are ectopically expressed from the same promoter and the extreme N-terminal deletions (Δ1-66, Δ1-25 Fig. 7D) resulted in protein levels comparable to WT, this suggests that sequences between 102 and 139 are particularly important for this effect. Also, while all the low expressing mutants are predominantly nuclear, localization of RanBPM in the nucleus is likely not the cause of the reduced expression, as other mutants comprising domain deletions that re-localize RanBPM to the nucleus, such as Δ212 and Δ360 are expressed at levels comparable to WT (Fig. 7E). Finally, point mutations in either NES or NLS2 did not affect expression (Fig. 7E). A summary of level of expression and subcellular localization of the mutants tested in this study is shown in Table 1.

**Table 1:** Summary of protein expression and subcellular localization for RanBPM mutants.

Mutant	Expression	Subcellular Localization
WT	***	N = C
ΔC1	***	N = C
ΔC2	***	N = C
ΔC3	***	N = C
ΔC4	***	N = C
ΔN1	*	N>C
ΔN2	**	C>N
ΔN3	*	N>C
ΔN4	*	N>C
Δ1-66	***	C>N
Δ1-25	***	C>N
Δ212	***	N>C
Δ360	***	N>C
ΔLisH	***	N = C
ΔCTLH	***	N>C
NLS1 Mut (150, 152, 153)	***	N>C
NES Mut (147, 151)	***	N>C
NLS2 Mut (635, 636, 640)	***	N = C

*low, **medium ***high expression, N = C nucleocytoplasmic, N>C mostly nuclear, C>N mostly cytoplasmic

Examination of the N-terminal region revealed the presence of two putative USP7 (ubiquitin-specific protease 7) binding sites at residues 39–42 and 125–128. USP7, also known as HAUSP (herpes virus associated ubiquitin-specific protease), is a member of the superfamily of deubiquitinating enzymes that are responsible for the removal of ubiquitin from their target proteins [41]. USP7 was previously shown to interact with and stabilize p53 and Mdm2 [42]. USP7 interacts with p53 at two closely spaced USP7 binding sites and both sites are needed for USP7 binding [43]. Therefore USP7 N-terminal sites appeared to be good candidates to regulate RanBPM stability. One or both USP7 sites were mutated by site-directed mutagenesis resulting in point mutations or deletion of the USP7 binding consensus sequences, and protein expression was assessed by western blot analysis (S3 Fig.). Since all resulting mutants were expressed at levels similar to that of wild-type, we concluded that the USP7 sites do not regulate RanBPM protein expression.

Next, we examined WW binding motifs present at positions 17–22 and 117–122 of RanBPM. Binding motifs of class IV WW domains such as the ones found at these positions are characterized by two proline residues and a phosphorylated serine/threonine residue [44]. This particular motif was chosen because class IV WW motifs have been shown to be a target of the peptidyl-prolyl cis-trans isomerase Pin-1 and isomerization of these WW motifs is key as kinases, phosphatases and ubiquitin ligases specifically recognize the cis or trans conformation of the prolyl peptide bond of their substrate, ultimately resulting in modulation of protein stability [45,46]. Mutation of critical residues within both WW motifs resulted in protein expression similar to that of WT RanBPM (S3 Fig.), suggesting that these WW motifs do not affect RanBPM protein expression.

### RanBPM is associated with microtubules in the cytoplasm and with chromatin in the nucleus

**Fig 8:**
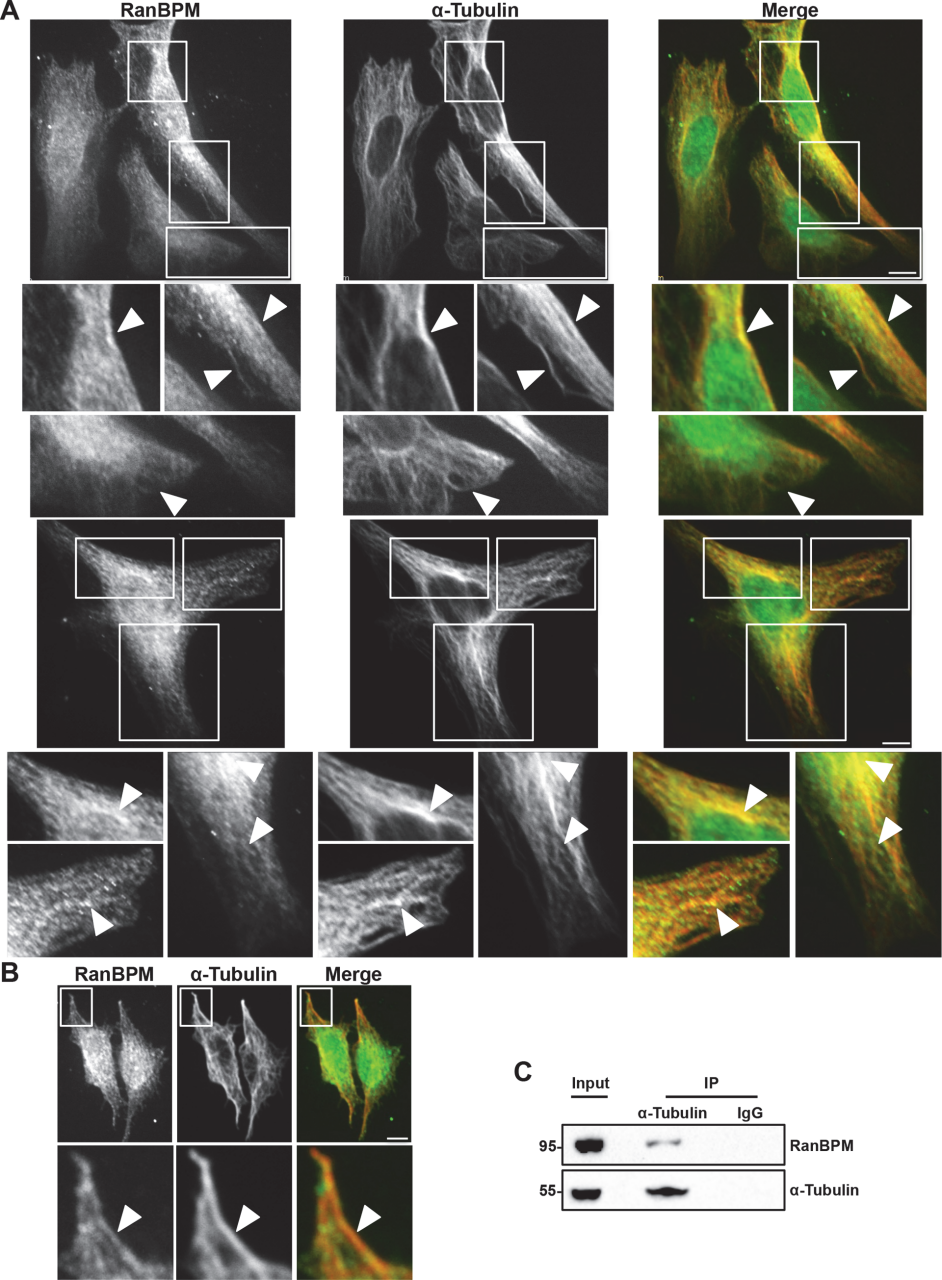
RanBPM is associated with microtubules. **A)** Hela and **B)** 3T3 MEFs were fixed and incubated with antibodies against RanBPM and α-tubulin. Shown are single plane confocal images. Insets are enlarged images of the boxed regions from the above panels and arrows indicate areas of colocalization. The right panels show merged images (RanBPM, green; α-tubulin, red). Scale bar: 10μm. **C)** Hela whole cell extracts were incubated with either an α-tubulin antibody or mouse IgG control. Immunoprecipitates were analyzed by western blot using RanBPM and α-tubulin antibodies and compared with 5% of input proteins.

We next investigated whether RanBPM is associated with particular structures in the nucleus and in the cytoplasm. Several lines of evidence suggest that RanBPM may be associated with microtubules. First, the LisH/CTLH domain is a domain present in proteins that are associated with microtubules [8,9]. In addition, RanBPM was reported to cofractionate with components of microtubules, such as dynactin and dynein [47]. Moreover, our findings that the LisH/CTLH domain can promote cytoplasmic localization of a nuclear protein and that deletion of the RanBPM LisH/CTLH domain results in its nuclear accumulation suggested that this domain could be mediating cytoplasmic retention through microtubule binding. Thus we investigated whether RanBPM colocalizes with α-tubulin, a main component of microtubules [48] using confocal microscopy analyses of endogenous RanBPM and α-tubulin (Fig. 8). RanBPM displayed a punctate pattern throughout the cytoplasm which indeed revealed partial colocalization with α-tubulin at specific microtubule structures in both Hela (Fig. 8A) and 3T3 mouse embryonic fibroblasts (MEFs, Fig. 8B). To substantiate the colocalization of RanBPM with α-tubulin, we performed co-immunoprecipitation experiments which revealed that endogenous RanBPM co-immunoprecipitated with endogenous α-tubulin (Fig. 8C). To determine whether microtubule depolymerization would affect RanBPM subcellular localization, we analyzed the effect of microtubule disruption on RanBPM subcellular localization. Treatment with nocodazole, which interferes with microtubule polymerization, surprisingly did not alter the subcellular localization of RanBPM (data not shown), indicating that RanBPM cytoplasmic localization is not sensitive to microtubule depolymerization. Overall, these data show that cytoplasmic RanBPM is partially associated with microtubules and suggest that RanBPM cytoplasmic localization could be conferred, at least in part, through retention of RanBPM *via* microtubule interaction.

**Fig 9:**
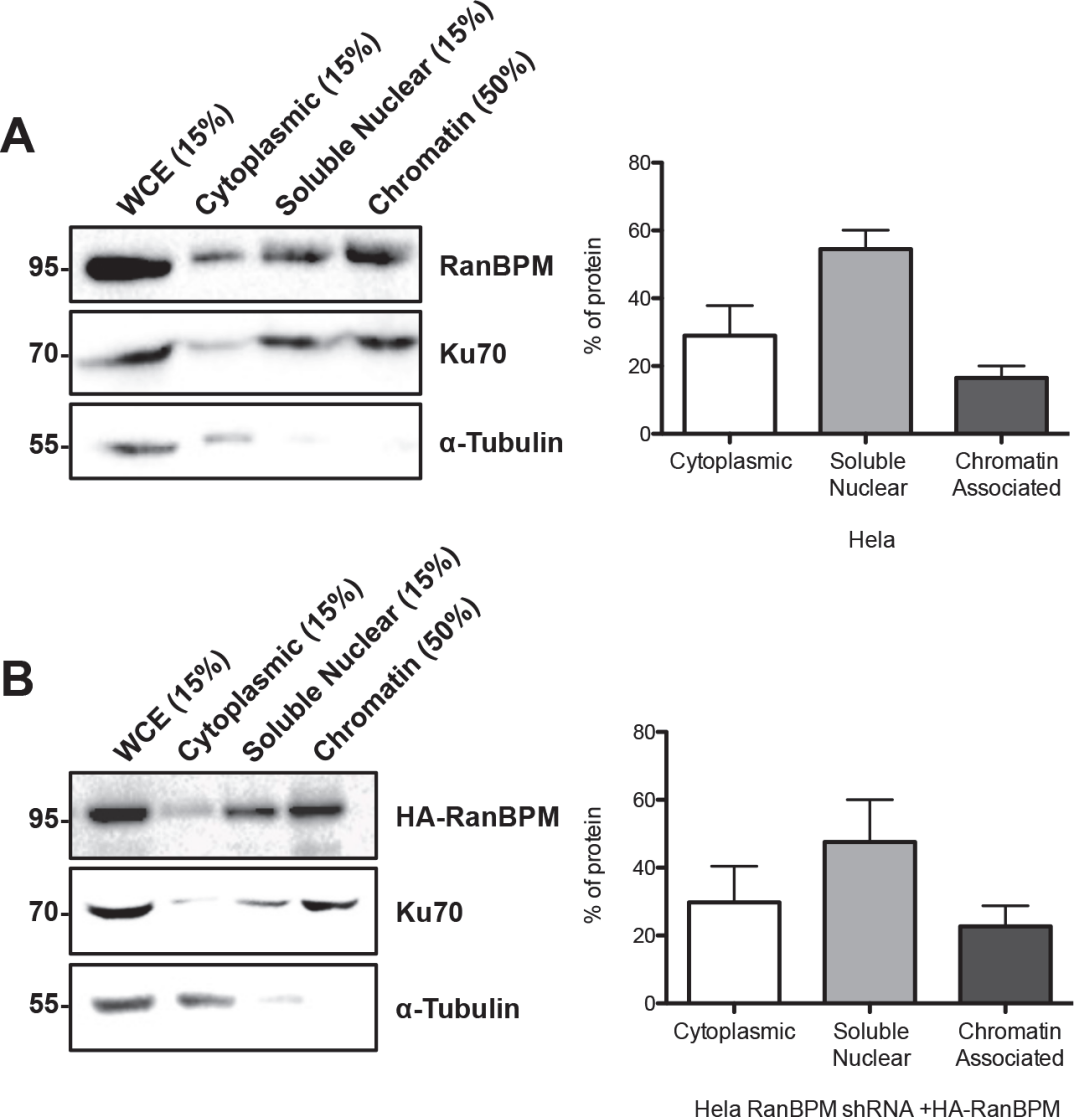
Quantitative analysis of RanBPM in cytoplasmic and nuclear fractions and its association with chromatin. **A)** Hela cell extracts were partitioned in cytoplasmic, nuclear soluble and chromatin fractions as described in Materials and Methods. Proportional amounts of each fraction were analyzed by western blot with the indicated antibodies. *Left*, representative western blot, with the percentage of each fraction loaded indicated above each lane. *Right*, bar graph representing the percentage of RanBPM protein present in each fraction. Data represent averages from three separate experiments with error bar representing SD. **B)** Hela RanBPM shRNA cells were transfected with pCMV-HA-WT-RanBPM and processed as in A.

To assess the status of RanBPM in the nucleus, we performed subcellular fractionations. Consistent with our immunofluorescence evaluations and previous analyses [22,29], quantification of endogenous RanBPM in Hela cytoplasmic and nuclear fractions showed that about 70% of RanBPM was present in the nucleus versus 30% in the cytoplasm (Fig. 9A). While most of nuclear RanBPM was present in the nuclear soluble fraction, approximately 20% of RanBPM was detected in the chromatin fraction, suggesting its association with DNA. We obtained identical results with ectopically expressed HA-RanBPM in Hela RanBPM shRNA cells (Fig. 9B). Overall, these results suggest that RanBPM can associate with microtubules in the cytoplasm and with chromatin in the nucleus.

## Discussion

Determining the physiological role played by RanBPM and its associated CTLH complex requires a detailed understanding of how its subcellular localization is regulated and contributes to its activity. In this study, we have investigated the contribution of various RanBPM protein regions to its subcellular localization using RanBPM shRNA cells, which allowed the analysis of ectopically expressed RanBPM mutants without interference from the endogenously expressed protein. Our results reveal that multiple regions and motifs regulate RanBPM nuclear and cytoplasmic localization and that in particular the RanBPM N-terminal domain is critical for both localization and protein stability.

### RanBPM determinants of nuclear localization

We determined that RanBPM possesses two motifs that confer nuclear localization: one sequence present at the extreme N-terminus (aa 1–25, hereafter called 1–25 poly-P/Q motif), and a non-canonical NLS located near the C-terminus (NLS2, Fig. 4). The 1–25 poly-P/Q motif functions as a NLS as it was able to promote nuclear localization of a cytoplasmic GFP-β-gal fusion construct. This region contains two proline stretches flanking a series of glutamines (S4 Fig.), but, to our knowledge, does not contain any sequence matching previously characterized NLS consensus. The most likely possibility is that this region interacts with a partner protein that promotes its translocation to the nucleus. A search for conserved motifs indicates that the 1–25 poly-P/Q motif contains four non-canonical class I-type SH3 binding motifs, and one overlapping WW class IV Peptidyl-Prolyl Isomerase (Pin1) binding motif. Future investigations will be needed to identify protein(s) interacting with this region. It is worth noting that Pin1 has been shown to promote nuclear localization of Rel proteins [49] and the adenosine deaminase ADAR2 [50], however how this is achieved remains unknown.

The RanBPM aa 635–649 sequence (NLS2) appeared to present some of the characteristics of a bi-partite NLS, with two clusters of basic amino acids. However, while mutations in the first cluster (R635A, R656A and K640A) did inhibit its ability to direct cytoplasmic GFP-β-gal to the nucleus, surprisingly, alanine substitutions of the three lysines in the second cluster of the motif (K645A, K646A and K649V) did not prevent NLS activity (data not shown). This suggests that NLS2 activity is dependent on determinants present in the first cluster of basic residues, in the sequence “RRDCGK”. Interestingly, this motif has similarities to that of a non-canonical NLS recently identified in a BRCA1 splice variant (KRAAER) [51], with two basic residues separated from a third basic residue by three amino acids.

Both the 1–25 poly-P/Q motif and NLS2 elements were able to direct a cytoplasmic GFP-β-gal construct to the nucleus. However, our results suggest that RanBPM nuclear localization is primarily dependent on the 1–25 poly-P/Q motif and that the C-terminal NLS2 does not appreciably contribute to RanBPM localization in normally cycling cells, conditions used in our experiments. This conclusion is supported by two observations: first, deletion of the C-terminal region containing NLS2 and mutation of the NLS2 sequence had little effect on RanBPM subcellular localization, and second, deletion of aa 1–25 poly-P/Q motif prevented RanBPM nuclear localization. One possibility to explain why NLS2 is not functional in the context of the full length RanBPM protein is that it is masked by protein folding. Indeed, we observed a dramatic shift in RanBPM localization from cytoplasmic to nuclear upon deletion of the 102–138 region (in the ΔN3 mutant) compared to the ΔN2 mutant. Thus, we postulate that this region normally folds over RanBPM C-terminus and masks NLS2, and that its deletion relieves the inhibition on NLS2. This folding could serve to modulate the activity of sequences such as NLS2 that are present in the C-terminal region in response to stress or physiological stimuli. A previous study identified this element as a putative NLS [37]. This study also reported that an N-terminal proteolytic fragment of RanBPM (aa 1–392) displayed cytoplasmic localization and this was attributed to the loss of that element in the C-terminal region of the protein. Since we have demonstrated that NLS2 is not imperative for nuclear localization, this is unlikely to be the case, however the reason for the cytoplasmic localization of this proteolytic fragment remains to be elucidated.

Our analysis showed that about 20% of total RanBPM, which represents about a third of the RanBPM nuclear pool, is associated with chromatin. RanBPM does not appear to comprise sequences conferring DNA binding properties, thus we postulate that its association with chromatin is mediated through interaction(s) with chromatin-associated partners. RanBPM was previously reported to interact with the Transcription Factor IID (TFIID) subunit TAF4 and with the glucocorticoid, androgen and thyroid receptors [11,13,14]. The thyroid receptor was suggested to interact with the C-terminal region of RanBPM [13], but the localization of the complex was not evaluated. Interestingly, the interaction of p73 with RanBPM was shown to promote RanBPM localization to the nucleus [12]. Whether and how RanBPM affects genomic regulations and DNA metabolism and whether this is in the context of the CTLH complex or in association with other proteins will need to be investigated.

### RanBPM in the cytoplasm

We have identified three motifs or domains that promote cytoplasmic localization: a *bona fide* NES, the SPRY domain and the LisH/CTLH domain. RanBPM NES presents the characteristics of a typical leucine-rich motif [52], but we also noted the presence of basic amino acids which led us to hypothesize that it may also function as a NLS. However, this element was only able to direct cytoplasmic localization of a nuclear GFP-β-gal fusion construct, suggesting that it only functions as a NES. We showed that this NES is readily sensitive to LMB in isolation (GFP-β-gal) which is further typical of leucine-rich NES which are CRM1/Exportin1-dependent [2,52]. But, while mutation of the NES promoted RanBPM nuclear accumulation, endogenous RanBPM was only sensitive to LMB treatment when subjected to LMB for longer periods of time, suggesting that only a small fraction of RanBPM is actively shuttling. In parallel, we found that deletion of both SPRY and LisH/CTLH domains promoted RanBPM nuclear accumulation. Since neither domain contains any identifiable NES, cytoplasmic localization through these domains most likely occurs through protein-protein interactions. As we have shown that RanBPM is associated with microtubules, it is possible that the LisH/CTLH domain could mediate RanBPM recruitment to microtubules and that this serves to retain RanBPM in the cytoplasm. As for SPRY domains, they are found in a wide array of proteins and are known to engage in protein-protein interactions. SPRY domain-containing proteins have been suggested to function as adaptors and play roles as scaffold proteins in a variety of signaling pathways [6]. Several proteins have been shown to interact with RanBPM through the SPRY domain in both the nuclear and cytoplasmic compartments. In the nucleus RanBPM has been shown to interact with cyclin-dependent kinase 11 CDK11(p46) [53], the immediate-early protein Rta of Epstein-Barr virus [54], and the ubiquitin-specific protease USP11 [55]. The SPRY domain of RanBPM has been demonstrated to interact with cytoplasmic or membrane bound proteins such as the TNF receptor associated factor TRAF6 [56], the receptor tyrosine kinase MET [57], the neural cell adhesion molecule L1 [58], and the neurotrophin receptor TrkA [59]. However, the contribution of these interactions to RanBPM subcellular localization was not investigated. Our results imply that the SPRY domain functions as a cytoplasmic restraint, suggesting that it mediates interaction of RanBPM with cytoplasmic protein(s), although this remains to be confirmed.

RanBPM has long been suspected to associate with microtubules, and was previously reported to cofractionate with components of the microtubules dynein and dynactin and dynamitin [47]. We show here that RanBPM indeed colocalizes and associates with α-tubulin. However, it is not clear whether this reflects a direct interaction with microtubule components, or if it is due to RanBPM association with microtubule-interacting proteins. Studies of the microtubule motor-regulating protein LIS1, the most extensively studied LisH-containing protein, suggest that the N-terminal LisH domain of LIS1, which is necessary for microtubule association, is not involved in dynein binding (which occurs through the C-terminal of LIS1), but that dimerization of LIS1 through the LisH domain is essential for dynein motility [60]. Therefore, we speculate that RanBPM is associating with microtubules through the LisH domain, however this remains to be determined. Interestingly, we recently reported that RanBPM forms a complex with the histone deacetylase HDAC6 [30]. HDAC6 is a microtubule-associated deacetylase that regulates α-tubulin acetylation and participates in microtubule metabolism [61–63], so the possibility exists that RanBPM is recruited to microtubules through HDAC6.

### Regulatory function of the N-terminal domain

RanBPM N-terminal region has a dual function in regulating RanBPM subcellular localization as it harbours both nuclear targeting and nuclear export sequences. Interestingly, in addition to modulating subcellular localization, the N-terminal 102–139 region also appears to affect protein stability. Progressive deletion of the N-terminal sequences resulted in a gradual decrease in protein expression, most notably when deleting the 102–139 region (ΔN3). Mutations in this region (WW and USP7 motifs) did not affect protein stability, suggesting that it is not the sequences *per se* that are important but that the region may function intramolecularly to regulate stability and localization.

One possibility to explain the changes in protein stability and subcellular localization observed with N-terminal deletions is that the RanBPM C-terminus is unstable/unfolded in absence of the N-terminal region. In support of this, a previous study documented that C-terminal fragments of RanBPM (corresponding to the C-terminal 350 aa) were very weakly expressed when transfected in mammalian cells [15]. RanBPM N-terminal region contains several amino acid repeats (proline, glutamine, alanine) that are characteristic of a low complexity regions (LCR) predicted to be unstructured [64]. LCRs are often found in ‘hub’ proteins and C-terminal LCRs have been predicted to have high levels of connectivity and are enriched in stress-response proteins [65]. While the function of the RanBPM LCR is unknown, our data suggest that it is critical for RanBPM stability and subcellular localization.

It is puzzling is that the deletion of the 102–139 (ΔN3) results in significant accumulation of RanBPM in the nucleus, despite the presence of the NES and the central domains, SPRY and LisH/CTLH, which would be expected to collectively allow cytoplasmic localization. Moreover, removal of the NES in the ΔN4 mutant and further deletion encompassing part of the SPRY domain (ΔN1) only marginally increased nuclear localization. Since this prominent nuclear localization occurs concurrently with the decrease in the level of protein expression, it is possible that the deletion of the N-terminal domain promotes the degradation of the cytoplasmic pool of these mutants, leaving the nuclear fraction somewhat stable.

RanBPM is well conserved between eukaryotic species, with the SPRY, LisH, CTLH and CRA domains being conserved throughout eukaryotes [28]. Both the NES identified within residues 140 and 155 and NLS2 comprising residues 635 and 649 are well conserved in mammals and in chordates in general, while in arthropods, such as Drosophila, these elements are partially conserved. The N-terminal domain however is the least conserved region of the protein. In particular, the poly-Q and poly-P repeats present in the human RanBPM N-terminus are not found in most homologs. Therefore the RanBPM 1–25 poly-P/Q motif is not present in other species, except in the mouse homolog where it is partially conserved [66]. Thus the regulations conferred by the 1–25 poly-P/Q region and the N-terminal region in general may only occur in human and possibly in mouse RanBPM, perhaps allowing NLS2 to be the predominant NLS in other species. In the *S*. *cerevisiae* RanBPM homolog Gid1, the domains are conserved however the NLS/NES motifs that we identified are not conserved.

Finally, one major element that may be contributing to RanBPM localization is its interaction with members of the CTLH complex. All components of the CTLH complex have been shown to be present within both the nuclear and cytoplasmic compartments with the exception of MAEA (Macrophage Erythroblast Attacher, also called p48EMLP, or EMP), which is only present in the nucleus and muskelin, which is mostly cytoplasmic [15,22]. These two proteins have been demonstrated to influence the localization of the other complex components. Ectopic expression of MAEA was shown to trigger increased recruitment of Twa1, the armadillo-repeat protein ARMC8α and RanBPM to the nucleus [22]. MAEA has been shown to contain a putative NLS between aa 110–113, however whether this element is a functional NLS that imports MAEA to the nucleus has not been determined [67]. Conversely, overexpression of muskelin resulted in cytoplasmic localization of Twa1, ARMC8α and RanBPM [22]. Previous analyses suggested that the subcellular distribution of muskelin is also modulated by several domains, a C-terminal domain that restrains it in the cytoplasm and a LisH domain that, contrary to that of RanBPM, has nuclear targeting activity [15]. However, the details of the CTLH complex formation remain unclear and the effect of RanBPM localization on the other members of the CTLH complex remains to be elucidated.

In yeast, RanBPM (Gid1) was found to be a crucial component for the architecture of the Gid complex as any alteration in this protein was found to disrupt the complex [25]. Given the high conservation of the members of the complex between yeast and mammals, it will be interesting to determine how the RanBPM mutations that affect its subcellular localization influence the CTLH complex formation and localization.

## Supporting Information

S1 Fig Validation of subcellular localization scoring protocol. **A)** Cells were fixed 24h after transfection of the RanBPM mutants indicated and incubated with an HA antibody and then with an Alexa Fluor 555 secondary antibody. Nuclei were stained with DAPI. Subcellular localization was quantified with ImageJ as described in materials and methods. Data represent averages from three separate experiments, each assessing approximately 100 cells. Error bars represent standard error. Mutant RanBPM constructs versus WT, ***, *P*<0.001; **, *P*<0.01; *, *P*<0.05. **B)** Cells from two clonal derivatives, Hela RanBPM shRNA 2–7 (employed throughout the study) or Hela RanBPM shRNA 2–6 were fixed 24h after transfection of the RanBPM mutants indicated and incubated with an HA antibody and then with an Alexa Fluor 555 secondary antibody. Nuclei were stained with DAPI. Subcellular localization was scored as either, N>C (nuclear greater than cytoplasmic), N = C (nuclear equal to cytoplasmic), or C>N (cytoplasmic greater than nuclear). Data represent averages from three separate experiments, each assessing approximately 100 cells. Error bars represent SD. Statistical analysis was performed to compare RanBPM shRNA clone 2–7 versus RanBPM shRNA clone 2–6 for each RanBPM mutant construct.
(PDF)

S2 Fig Effect of short LMB treatment on RanBPM nuclear export. **A)** Hela cells treated with EtOH or 20nM LMB were fixed 3h after treatment. Cells were processed for immunostaining with antibodies to RanBPM and cyclin B1 and nuclei stained with DAPI. At least 100 cells were scored as N>C (nuclear greater than cytoplasmic), N = C (nuclear equal to cytoplasmic), or C>N (cytoplasmic greater than nuclear). Data represent averages from three separate experiments. Error bars represent SD. **B)** RanBPM shRNA Hela cells transfected with pCMV-HA-WT-RanBPM were incubated O/N and treated with EtOH or 20nM LMB for 3h. Cells were analyzed as described above. Scale bar: 10μm.
(PDF)

S3 Fig Mutations of N-terminal USP and WW motifs do not affect RanBPM expression. **A)** Amino acid sequence and position of the USP and WW domains in RanBPM. Mutations are indicated to the right. The predicted motifs are underlined and mutations are marked in red and deletions are represented by a dash (-). USP7 1Δ and 2Δ mutant is comprised of both 1Δ and 2Δ mutations. **B)** Whole cell extracts were prepared from RanBPM shRNA Hela cells transfected with pCMV-HA-RanBPM mutant constructs 24h after transfection. An HA antibody was used to detect HA-RanBPM and β-actin was used as a loading control. Western blots show expression of WT and USP mutant constructs. **C)** Expression of WT and WW mutant constructs as described in B.
(PDF)

S4 Fig Amino acid 1–25 of RanBPM. The sequence of the first 25 amino acids of human RanBPM is shown.
(PDF)
