# Supplementary figures and images for: Characterization of RanBPM Molecular Determinants that Control Its Subcellular Localization
Source: PLoS One. 2015 Feb 6;10(2):e0117655. doi: 10.1371/journal.pone.0117655 (PMC4319831; doi:10.1371/journal.pone.0117655)

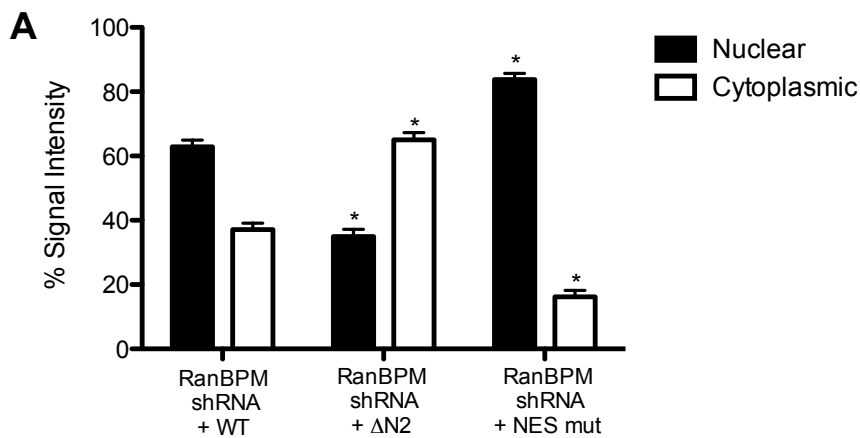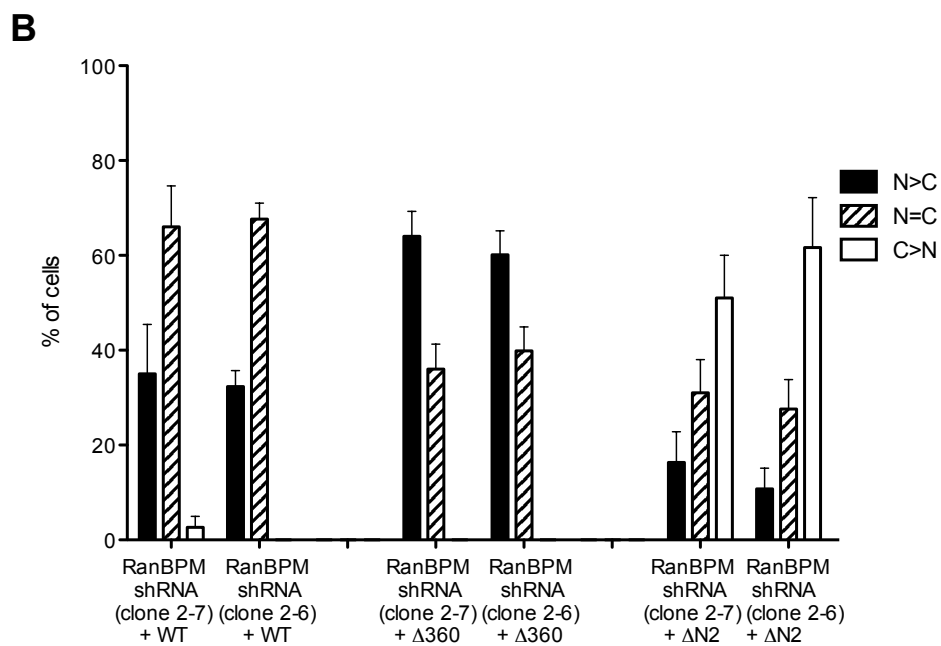

Supplement: S1 Fig — A) Cells were fixed 24h after transfection of the RanBPM mutants indicated and incubated with an HA antibody and then with an Alexa Fluor 555 secondary antibody. Nuclei were stained with DAPI. Subcellular localization was quantified with ImageJ as described in materials and methods. Data represent averages from three separate experiments, each assessing approximately 100 cells. Error bars represent standard error. Mutant RanBPM constructs versus WT, ***, P<0.001; **, P<0.01; *, P<0.05. B) Cells from two clonal derivatives, Hela RanBPM shRNA 2–7 (employed throughout the study) or Hela RanBPM shRNA 2–6 were fixed 24h after transfection of the RanBPM mutants indicated and incubated with an HA antibody and then with an Alexa Fluor 555 secondary antibody. Nuclei were stained with DAPI. Subcellular localization was scored as either, N>C (nuclear greater than cytoplasmic), N = C (nuclear equal to cytoplasmic), or C>N (cytoplasmic greater than nuclear). Data represent averages from three separate experiments, each assessing approximately 100 cells. Error bars represent SD. Statistical analysis was performed to compare RanBPM shRNA clone 2–7 versus RanBPM shRNA clone 2–6 for each RanBPM mutant construct. (PDF) [file pone.0117655.s001.PDF]

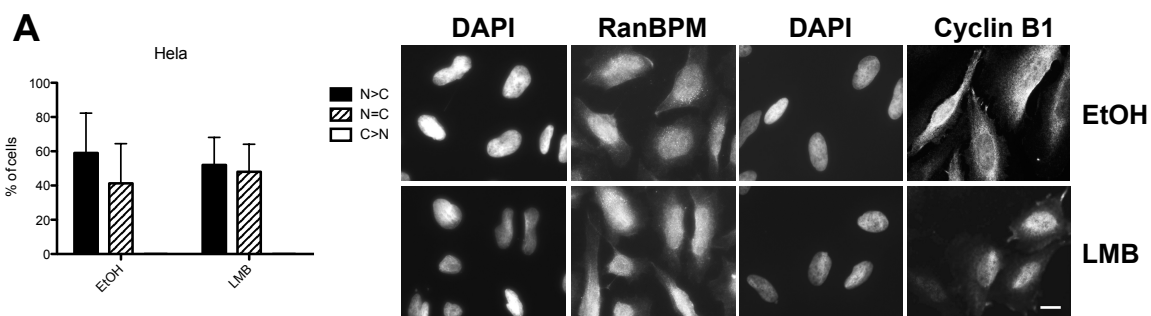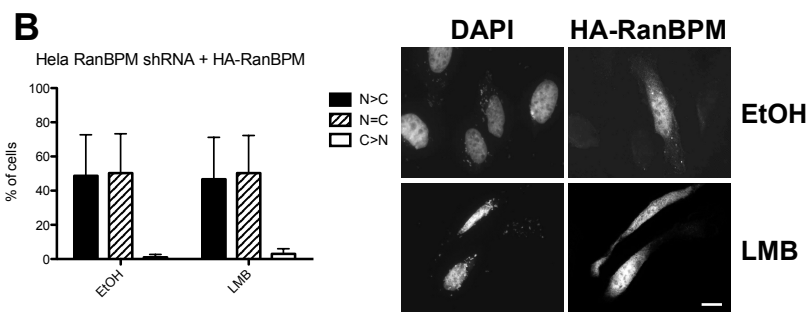

Supplement: S2 Fig — A) Hela cells treated with EtOH or 20nM LMB were fixed 3h after treatment. Cells were processed for immunostaining with antibodies to RanBPM and cyclin B1 and nuclei stained with DAPI. At least 100 cells were scored as N>C (nuclear greater than cytoplasmic), N = C (nuclear equal to cytoplasmic), or C>N (cytoplasmic greater than nuclear). Data represent averages from three separate experiments. Error bars represent SD. B) RanBPM shRNA Hela cells transfected with pCMV-HA-WT-RanBPM were incubated O/N and treated with EtOH or 20nM LMB for 3h. Cells were analyzed as described above. Scale bar: 10μm. (PDF) [file pone.0117655.s002.PDF]

**A**

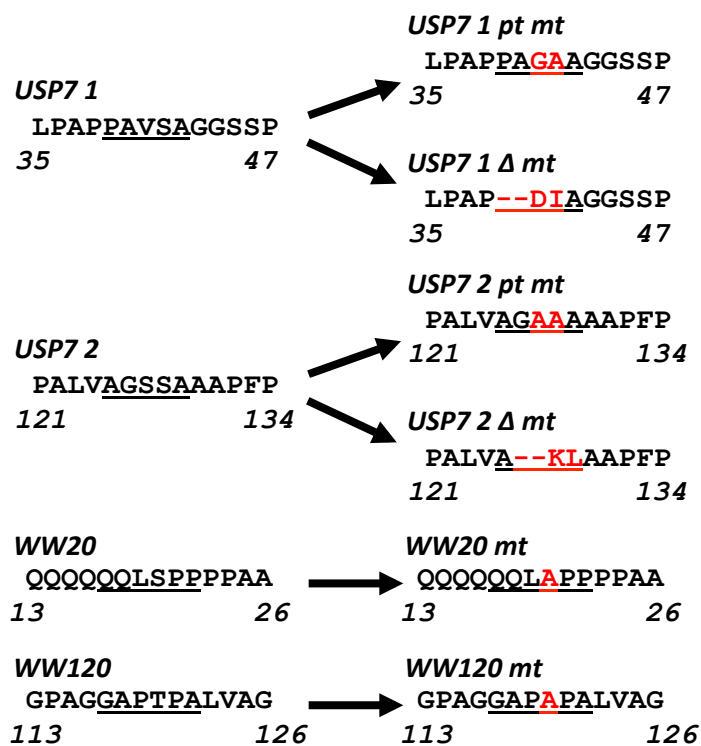

**B**

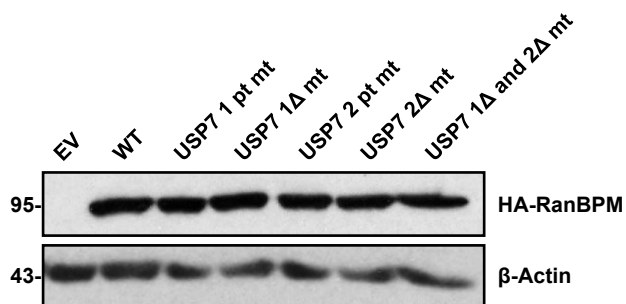

**C**

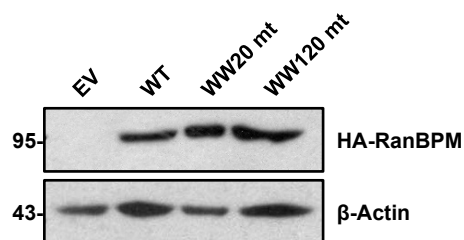

Supplement: S3 Fig — A) Amino acid sequence and position of the USP and WW domains in RanBPM. Mutations are indicated to the right. The predicted motifs are underlined and mutations are marked in red and deletions are represented by a dash (-). USP7 1Δ and 2Δ mutant is comprised of both 1Δ and 2Δ mutations. B) Whole cell extracts were prepared from RanBPM shRNA Hela cells transfected with pCMV-HA-RanBPM mutant constructs 24h after transfection. An HA antibody was used to detect HA-RanBPM and β-actin was used as a loading control. Western blots show expression of WT and USP mutant constructs. C) Expression of WT and WW mutant constructs as described in B. (PDF) [file pone.0117655.s003.PDF]
