# Supplementary material for: Characterization of RanBPM Molecular Determinants that Control Its Subcellular Localization
Source: PLoS One. 2015 Feb 6;10(2):e0117655. doi: 10.1371/journal.pone.0117655 (PMC4319831; doi:10.1371/journal.pone.0117655)
Supplement: S4 Fig — The sequence of the first 25 amino acids of human RanBPM is shown. (PDF) [file pone.0117655.s004.PDF]

MSGQPPPPPPQQQQQQQLSPPPPA

1 *25*

Suppl. Fig. S4
